# Nuclearly translocated insulin-like growth factor 1 receptor phosphorylates histone H3 at tyrosine 41 and induces *SNAI2* expression *via* Brg1 chromatin remodeling protein

**DOI:** 10.18632/oncotarget.9785

**Published:** 2016-06-02

**Authors:** Dudi Warsito, Yingbo Lin, Ann-Christin Gnirck, Bita Sehat, Olle Larsson

**Affiliations:** ^1^ Department of Oncology-Pathology, Cancer Center Karolinska, Karolinska Institutet, SE-171 76 Stockholm, Sweden

**Keywords:** IGF-1R, phosphorylation, histone, SNAI2, Brg1

## Abstract

The insulin-like growth factor-1 receptor (IGF-1R) is a receptor tyrosine kinase that has crucial roles in cell proliferation and protection from apoptosis. It is therefore not surprising that IGF-1R is often found overexpressed in many types of tumors. This has made IGF-1R a prominent target molecule for pharmacological companies to develop new anti-cancer agents. However, several clinical trials during the last 5 years using IGF-1R specific antibodies have shown disappointing results. We have previously shown that upon IGF-1 stimulation, the receptor becomes SUMOylated and translocates into the nucleus of cancer cells to act as a transcription co-factor. Soon after our original study, several others have reported nuclear IGF-1R (nIGF-1R) as well, and some of them have demonstrated a prognostic value of nIGF-1R expression in cancer. In the current study we demonstrate that nIGF-1R binds to and phosphorylates histone H3 at tyrosine 41 (H3Y41) in HeLa cells. Furthermore, our results suggest that phosphorylation of H3Y41 by nIGF-1R, stabilizes the binding of Brg1 chromatin remodeling protein to Histone H3. Our findings suggest that *phosphorylated* nIGF-1R, rather than total nIGF-1R, plays a superior role in these contexts. We identified *SNAI2* oncogene as a target gene for nIGF-1R and its expression was decreased upon mutation of H3Y41 or by Brg1 knockdown. Furthermore, we demonstrate that both IGF-1R and Brg1 binds to the *SNAI2* promoter. As SNAI2 protein is implicated in e.g. cancer invasion and metastasis, the nIGF-1R-mediated effects shown in this study may influence such important tumor phenotypic actions.

## INTRODUCTION

Posttranslational modifications of nucleosomal histones are essential for chromatin remodeling [1–3]. Of all the types of histone modifications that have been discovered, histone tyrosine phosphorylation took the longest [4]. In 2009, five studies concerning this modification were published. Phosphorylation of histone H2A.X at tyrosine 142 was shown to be involved in the decision of whether the cell undergoes DNA repair or apoptosis during DNA damage [5–7]. Singh et al. demonstrated that excess levels of histone H3 in yeast were phosphorylated at tyrosine 99, which resulted in polyubiquitinylation and proteasomal degradation [8]. Kouzarides lab provided evidence that histone H3 tyrosine 41 phosphorylation by Jak2 induced *lmo2* oncogene expression in leukemic cells [9].

Remodeling of the chromatin during transcription is a highly dynamic event and is achieved by the ordered recruitment of various chromatin binding proteins including the chromatin remodeling complexes [10, 11]. There are four families of chromatin remodeling complexes [10]. The remodeling activity of each complex is mediated by the ATP-dependent DNA helicase subunit. The SWI/SNF family of chromatin remodeling complexes was the first to be discovered and is a >1 MDa complex consisting of 10-12 subunits. The remodeling activity of the complex is facilitated by one of two ATP dependent helicases, Brg1 (Brahma-related gene 1) or Brm (Brahma) [12]. Many of the genes that SWI/SNF controls encode proteins important for cancer cell migration and invasion [13–16].

Signaling through the insulin-like growth factor 1 receptor (IGF-1R) leads to downstream activation of phosphatidylinositol 3′-kinase (PI3K)/Akt [17] and mitogen-activated protein kinase (MAPK)/Erk signaling pathways [18, 19], both of which promotes cell cycle progression and protection from apoptosis. IGF-1R is often found overexpressed in many human cancers [20, 21]. We recently demonstrated that IGF-1 stimulates the SUMOylation of IGF-1R at three evolutionary conserved lysine residues –K1025, K1100, and K1120 – in the β subunit of the receptor and induces nuclear translocation of the receptor [22]. Mutation of these lysine residues blocks SUMOylation of the receptor and reduces its accumulation in the nucleus without impairing its endocytosis or activation of Akt/Erk signaling pathways [22]. Aleksic et al. reported high levels of nuclear IGF-1R (nIGF-1R) in primary renal cancer cells and preinvasive lesions in the breast [23]. In renal cancer, presence of nIGF-1R was associated with poor prognosis.

The molecular mechanism by which nIGF-1R mediates its function is still an area to be explored. We recently showed that nIGF-1R is able to bind to putative enhancer sites in genomic DNA [22] and the cyclin D1 promoter [24] to activate transcription via LEF1 transcription factor. It was also shown that nIGF-1R binds histone H3 and RNA polymerase II [23] as well as to the IGF-1R promoter to induce its own expression [25]. These studies suggest that nIGF-1R could have a more direct role in activating its own target genes apart from its classical role at the plasma membrane.

In this study we aimed to investigate the possible functional interaction between nIGF-1R and H3 with special focus on transcriptional activation. We could show that nIGF-1R tyrosine phosphorylates histone H3 leading to stabilized Brg1-chromatin binding. This resulted in an increased expression of *SNAI2,* encoding the Snail zinc finger protein Snai2 that is involved in cancer invasion and metastasis.

## RESULTS

### General experimental strategies

To investigate the role of nIGF-1R we used two principally different experimental conditions; cells were grown either under basal conditions, i.e. in culture medium supplemented with 10% serum, which contains IGF-1 (5-15 ng/ml) and IGF-2 as well [26]; or were serum-starved and stimulated with IGF-1. The combined use of these conditions facilitates the understanding of nIGF-1R's functions. One benefit of basal conditions is that strong ligand-induced cell membrane IGF-1R signaling (e.g. Akt and Erk) is avoided, as over-signaling could mask specific functions of nIGF-1R. Also, basal conditions better resemble physiological ones, compared to external treatment with high concentration of ligand. In addition, the presence of serum makes the cells more resistant to stressful conditions like those used for transfections. On the other hand, IGF-1R activation due to external ligand supply has been shown to favor activity of the nuclear receptor [22, 23], making this strategy relevant. In a subset of experiments performed under basal conditions we explored the necessity of IGF-1R phosphorylation in mediation of nIGF-1R-mediated actions using NVP AEW-541 that is an IGF-1R-specific kinase inhibitor.

### IGF-1R binds to histone H3

Based on the finding of Aleksic et al. that histone H3 (H3) co-precipitates with nIGF-1R in DU145 prostate cancer cell lines [23], we made mass spectrometry on immunoprecipitated IGF-1R from nuclear extracts of human cervical carcinoma cells (HeLa). We could only detect a few proteins that co-precipitated with nIGF-1R in our analysis. But as histone H3 was one of them, we decided to investigate this interaction in further detail.

**Figure 1:**
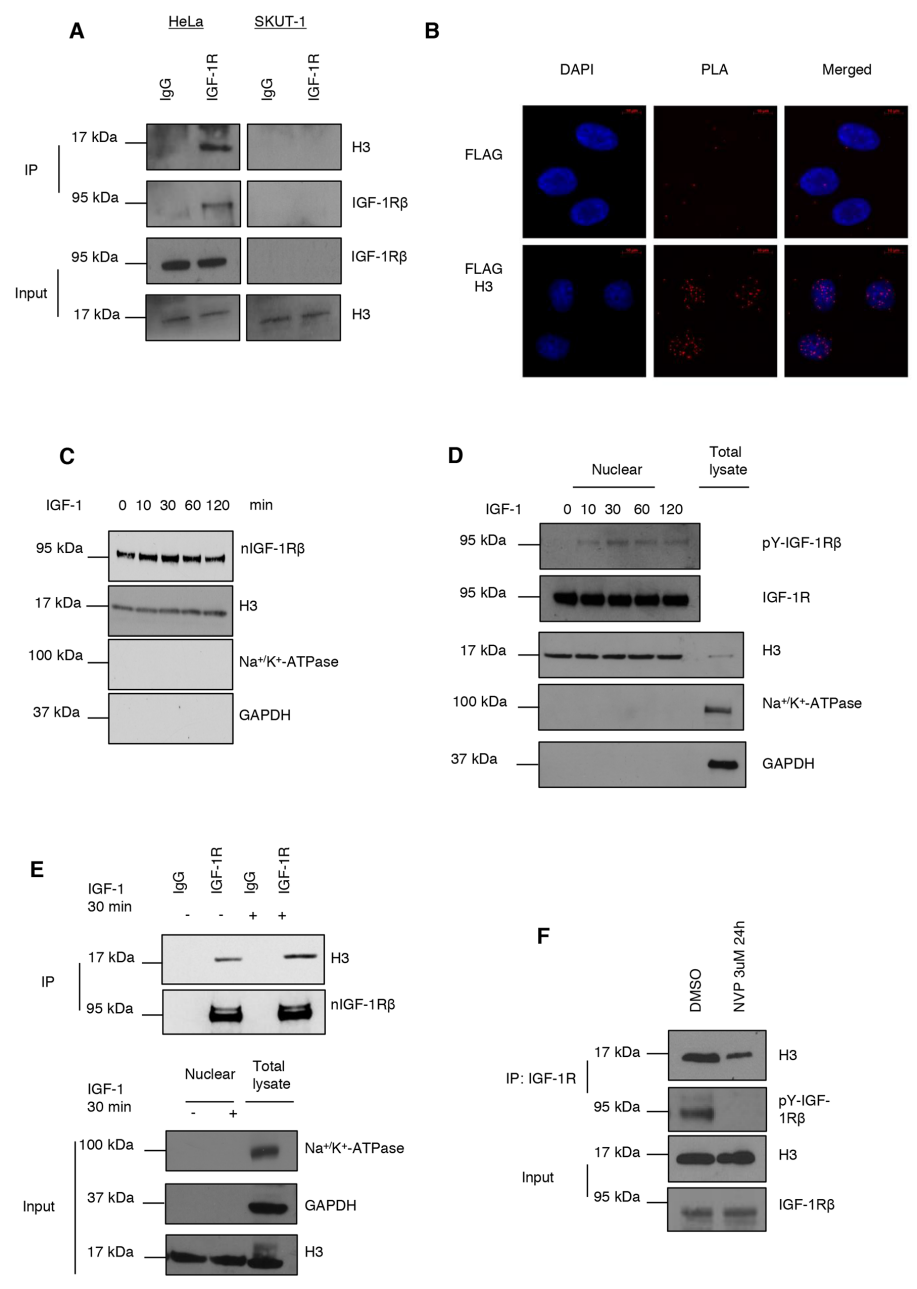
IGF-1R binds to histone H3 **A.** IGF-1R binding to histone H3 assayed by co-immunoprecipitation (co-IP). IP of IGF-1R and detection of H3 and IGF-1R, of total cell extracts from HeLa and IGF-1R deficient SKUT-1 cells (top panels). Cells were growing under basal conditions. IgG was used a negative IP control. Input samples are shown in bottom panels. **B.** nIGF-1R and histone H3 binding determined with PLA (red dots) in HeLa cells, growing under basal conditions, transfected with an empty Flag vector (top) or Flag H3 construct (bottom). Anti-Flag and anti-IGF-1R antibodies were used. **C.** Kinetics of nIGF-1R after 100 ng/ml IGF-1 stimulation (0-120 min) after 24 h serum starvation of HeLa cells. Nuclear extracts obtained by cell fractionation were analyzed for IGF-1R by western blotting Purity of nuclear fraction was determined by blotting for H3 (nucleus), Na^+^/K^+^-ATPase (membrane) and GAPDH (cytosol). **D.** Kinetics of phospho-nIGF-1R after IGF-1 stimulation. The experiment was performed as described in (C) with the exception that phosphorylated nIGF-1R, beside total IGF-1R, was detected using a specific phoshotyrosine-IGF-1R antibody. **E.** nIGF-1R binding to histone H3 after IGF-1 stimulation (30 min) in HeLa cells assayed by co-IP (IP with anti-IGF-1R and IgG) (top panels). Nuclear extracts were isolated and analyzed for H3 and IGF-1R. Purity of nuclear fraction was determined as in (C). **F.** Total cell extracts from HeLa cells, growing under basal conditions, treated with 3 μMIGF-1R tyrosine kinase inhibitor, NVP AEW-541, for 24 h were subjected to co-IP with IGF-1R antibody and blotted for histone H3 and tyrosine phosphorylated IGF-1R (top panels). Input samples (bottom panels).

After immunoprecipitation (IP) of cell lysates from HeLa using an IGF-1R antibody, and anti-IgG as control, we could confirm that IGF-1R binds to histone H3 by blotting with an H3 antibody (Figure 1A, left panel). The IgG control was negative. The specificity of the IGF-1R/H3 association was further supported by a negative outcome of co-IP in the IGF-1R deficient leiomyosarcoma cell line SKUT-1 (right panel of Figure 1A).

In order to determine how IGF-1R binds to histone H3 at a subcellular level, we generated and overexpressed a FLAG-tagged histone H3 construct in HeLa cells. A construct expressing only the FLAG-tag was used as negative control. Transfected cells were subjected to *in situ* Proximity Ligation Assay (PLA) [27] using IGF-1R and FLAG antibodies to determine any binding between IGF-1R and FLAG-H3. Figure 1B shows that IGF-1R binds to FLAG-H3 in the nucleus. We also tried investigating whether IGF-1R binds to endogenous histone H3 by PLA, but we did not get any clear signals in these experiments (data not shown). This might be explained by sterical hinders, e.g. H3 may be buried in a protein complex making it unavailable for the antibody. However, upon overexpression of FLAG-H3, nIGF-1R/H3 complexes were clearly detectable (Supplementary Figure S1). As a negative control we used an antibody to E2F1, which is not expected to bind to nIGF-1R. As shown there were no detectable nIGF-1R/E2F1 signals (Supplementary Figure S1).

### Ligand-induced phosphorylation of nIGF-1R and association with H3

Previous studies have shown that IGF-1R autophosphoryation is required for it to undergo nuclear translocation [22–24]. Therefore we now sought to investigate whether IGF-1R activation was required for IGF-1R to bind to H3 in HeLa cells. For this purpose we used two experimental conditions; IGF-1 stimulation of serum-starved cells or inhibition of IGF-1R tyrosine kinase activity in cells cultured in serum-enriched medium. We first investigated the kinetics for ligand-induced total nIGF-1R in HeLa cells, Figure 1C (top panel). The cells were serum starved for 24 h and stimulated with 100 ng/ml IGF-1 for 0-120 minutes. Nuclear proteins were extracted and blotted for IGF-1R. Our results demonstrate that IGF-1R accumulates slightly in the nucleus at 10 min and reaches its maximum at 30 minutes. Purity of the nuclear fraction was determined by blotting for H3 (nuclear), Na+/K+-ATPase (membrane) and GAPDH (cytosol).

Even if addition of the ligand increases nIGF-1R, the changes are minor, and it is noteworthy that the signal at time-point 0 (serum starvation control) is strong. The experiment was repeated several times with similar results. Similar pattern with high baseline and minor ligand-induced nIGF-1R increase was recently observed in serum starved non-small cell lung cancer cell (H1299) [24]. Also in the study by Aleksic et al, the baseline level of nIGF-1R remained quite high after serum-starvation [23].

Next we explored whether IGF-1 stimulation may increase the level of phosphorylated nIGF-1R. HeLa cells were serum-starved and stimulated with IGF-1 as described above. Levels of phospho-IGF-1R in the nuclear fraction were determined by western blotting using a phosphotyrosine-specific IGF-1R antibody (Y1135/Y1136). As shown in Figure 1D there was no detectable baseline phospho-nIGF-1R at time-point 0, whereas IGF-1 treatment caused a weak but clear phospho-nIGF-1R band that peaked at 30 min.

We also investigated if the same treatment regimen (serum-starvation followed by ligand stimulation) could increase the binding of nIGF-1R to H3, assayed by co-IP (Figure 1E). As demonstrated, there was a detectable baseline level of nIGF-1R/H3 complex in non-stimulated cells, but it increased after addition of IGF-1 for 30 min. This suggests that the ligand-induced increase in phospho-nIGF-1R is important for its binding to H3. The purity of the nuclear extracts is shown in bottom panel of Figure 1E. The next experiment shows that a specific IGF-1R kinase inhibitor (NVP AEW-541) abolished IGF-1R phosphorylation and strongly diminished IGF-1R binding to H3 (Figure 1F). Supplementary Figure S2 provides further evidence of the involvement of phosphorylated nIGF-1R. HeLa cells were transfected with an inactive IGF-1R (TM-IGF-1R), in which the three tyrosine sites (1131, 1135 and 1136) in the activation loop of the kinase domain are mutated. The co-IP experiment from nuclear extract confirms that TM-IGF-1R is not phosphorylated and that its binding to histone H3 is comparable with mock control. In contrast, the level of wt-IGF-1R/H3 is increased. Purity control is in right panel of Supplementary Figure S2.

### Evidence that IGF-1R phosphorylates H3

Having demonstrated that nIGF-1R binds to H3, we investigated the functional significance of this binding. Given the fact that IGF-1R harbors a tyrosine kinase domain, it was of special interest to investigate whether H3 might be a substrate of it. H3 contains three highly conserved tyrosine residues, Y41, Y54 and Y99. It was previously shown that histone H3 tyrosine 41 (H3Y41) could be phosphorylated by the non-receptor tyrosine kinase Jak2 and that this phosphorylation was enriched at promoters of highly active genes [9, 28].

**Figure 2:**
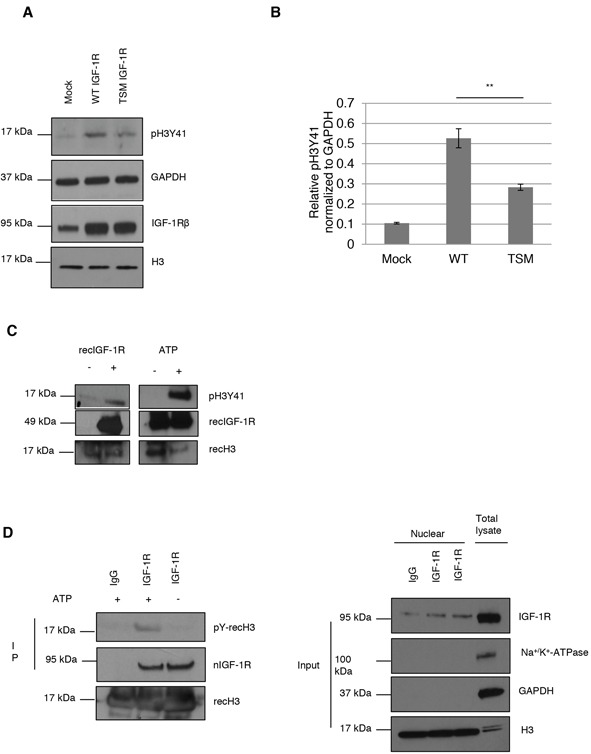
IGF-1R phosphorylates histone H3Y41 **A.** Histone H3Y41 phosphorylation was determined in HeLa cells, growing under basal conditions, transfected with mock (empy vector), *wt-IGF1R* or *tsm-IGF1R* (top panel). IGF-1R level (bottom panel) and GAPDH was used as loading control (middle panel). **B.** Quantification of H3Y41 phosphorylation. One-way ANOVA and Tukey's HSD test were perfomed. **p<0.01. Means and s.d. are shown (n=3) **C.** *In vitro* kinase assay using recombinant (rec) histone H3 and recombinant IGF-1R (amino acids 959-end) proteins. H3Y41 phosphorylation, and recIGF-1R and H3 as well, was assessed in absence or presence of recIGF-1R (Left panel). Right panel shows kinase assay with both proteins present but in the absence (negative control) or presence of ATP. **D.** *In vitro* kinase assay using endogenous nIGF-1R from HeLa cells growing under basal conditions. IgG and IGF-1R antibodies were used to immunoprecipitate nIGF-1R and kinase assay was performed as in (C). Tyrosine phosphorylated recombinant H3 was detected using a pan-phosphotyrosine antibody (left panels). Right panels shows purity of nuclear fractions.

Therefore we chose to focus on H3Y41. HeLa cells were transiently transfected with mock, *wt-IGF1R* or *tsm-IGF1R*. Whereas the wild type receptor (wt-IGF-1R) goes into the cell nucleus, the receptor with triple mutated SUMO binding sites (tsm-IGF-1R) exhibits a reduced nuclear translocation [22]. Cell lysates from these transfectants were analyzed for H3Y41 phosphorylation. As demonstrated, wt-IGF-1R increases phospho-H3Y41 (pH3Y41) 5-fold compared to mock, while tsm-IGF1R shows a 2.7-fold increase (Figures 2A and 2B). The latter result may be explained by that other signaling pathways are involved in phosphorylation of H3Y41. For example, it is known that IGF-1R can activate Jak2 signaling [29] and as mentioned above, Jak2 binds to and phosphorylates H3Y41. Alternatively, tsm-IGF-1R might undergo nuclear translocation to some extent through heterodimerizing with the insulin receptor, which can also be transported to the cell nucleus [25]. Anyway, we can conclude that *wt-IGF1R* transfected cells exhibit a significantly higher H3Y41 phosphorylation compared to tsm-IGF1R transfected ones.

In order to determine whether IGF-1R in itself serves as the kinase for H3Y41 phosphorylation we performed an *in vitro* kinase assay using recombinant hH3 and hIGF-1R kinase (amino acids 959-end). In the absence of recombinant IGF-1R, H3Y41 phosphorylation could not be detected (Figure 2C). Addition of recombinant IGF-1R induced H3Y41 phosphorylation (Figure 2C). The right panel of Figure 2C confirms that H3Y41 phosphorylation does not occur in the absence of ATP (negative control) in the reaction tube. We further studied IGF-1R kinase activity by using endogenous IGF-1R. nIGF-1R from HeLa cells was immunoprecipitated with IgG or IGF-1R antibodies. Immunoprecipitated nIGF-1R was incubated with recombinant histone H3 in presence or absence of ATP. Tyrosine phosphorylation of histone H3 was analyzed by western blot using a pan-phosphotyrosine antibody since the pH3Y41 specific antibody had been discontinued. Our result shows that in presence of ATP, endogenous IGF-1R is able to phosphorylate histone H3 (left panel Figure 2D). Right panels of Figure 2D show purity of the nuclear fraction. Together, these data suggest that nIGF-1R binds to H3 and phosphorylates it at tyrosine 41.

### Absence of H3Y41 destabilizes Brg1-histone H3 association

Access to the DNA by the transcriptional machinery is achieved by the recruitment of co-factors and chromatin remodeling complexes to histones. Many of these protein complexes harbor intrinsic protein binding domains that recognize specific protein modifications. Thus, modified histones set a platform for these complexes. Lavigne et al. demonstrated that Brg1 chromatin remodeling factor binds histone H3 at a region close to Y41 to remodel chromatin *in vitro* [30]. Based on these results and the finding that phosphorylation of H3Y41 induces transcription [9], we focused on this specific modification in our attempts to investigate the possible recruitment of Brg1 to histone H3.

**Figure 3:**
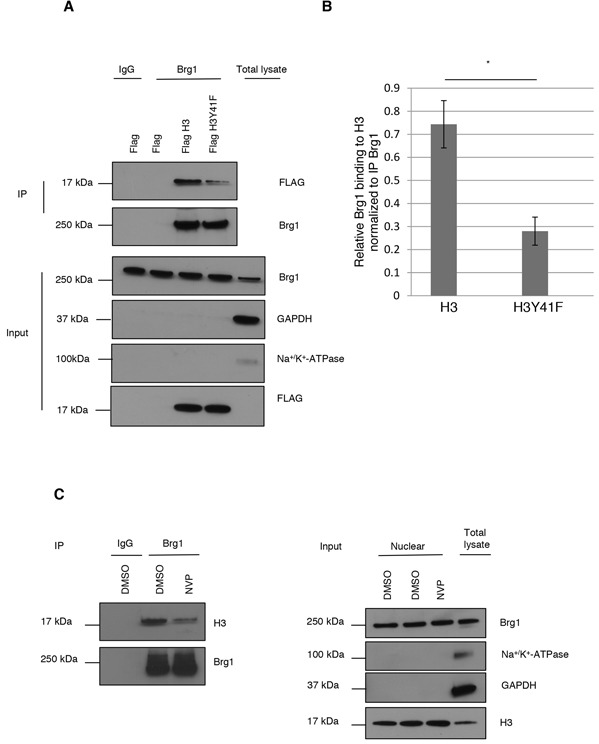
pH3Y41 potentially stabilizes Brg1 binding to chromatin **A.** Nuclear extract from HeLa cells, cultured under basal conditions, transfected with Flag, Flag H3 or Flag H3Y41F mutant constructs were subjected to immunoprecipitation with IgG or Brg1 antibodies and blotted for Flag and Brg1 (top panels). Input samples (bottom panels). **B.** Quantification of Brg1 binding to histone H3. Level (relative units) of Brg1 was normalized to level of IP Brg1. Two-sided Student's t-test. *p<0.05. Means and s.d. are shown (n=3). **C.** HeLa cells, cultured under basal conditions, were treated with DMSO or 3 μM NVP for 24 h. Nuclear extracts were subjected to IP with IgG or Brg1 antibodies and blotted for histone H3 and Brg1 (left panels). Right panels shows purity of nuclear fraction.

To evaluate if pH3Y41 could increase binding of Brg1 to H3, we generated a Flag-tagged wild type H3 and a H3Y41F phospho-mutant construct. HeLa cells were transiently transfected with empty vector or either of the H3 constructs. Nuclear lysates were subjected to IP of Brg1 and blotted for Flag. Figure 3A and 3B shows that binding of Brg1 to H3Y41F was reduced compared to wild type H3. As H3Y41F does not fully block Brg1 binding, it is probable that Y41 phosphorylation *per se* is not necessary for Brg1 to bind H3, but it may stabilize it.

To investigate if IGF-1R tyrosine kinase activity was of importance for Brg1 binding to H3, HeLa cells were treated with NVP AEW-541 for 24 h. After isolation of nuclear extracts, IP of Brg1 with detection of H3 and Brg1 was run. IgG was used as a negative IP control. As shown in Figure 3C, inhibition of IGF-1R phosphorylation reduced Brg1 binding to H3. Right panel shows purity control.

Taken together, our results suggest that nIGF-1R-induced phosphorylation of H3Y41 contributes to recruitment of Brg1 to H3.

### SNAI2 expression is increased by nIGF-1R and H3

In order to identify genes whose expression are induced by nIGF-1R and pH3Y41, we first performed a qPCR array using the RT^2^ Profiler PCR Array kit from Qiagen (Hilden, Germany). HeLa cells transfected with *wt-IGF1R* or *tsm-IGF1R* were used. The cells were cultured under basal conditions as we wanted to decrease the influence of IGF-1 stimulated membrane IGF-1R and its canonical signaling pathways as much as possible. Supplementary Table S1 demonstrates a large number of genes whose expression differed between *wt-IGF1R* or *tsm-IGF1R*. Only genes that encode nuclear or nuclear-associated proteins are shown in Supplementary Table S1. The selection of a gene for further studies was based on any known connection with IGF-1R signaling. Herewith we found *SNAI2*, upregulated by *wt-IGF1R* expression (Supplementary Table S1), to be of interest. Snai2 belongs to the Snail family of zinc finger proteins that promotes epithelial-mesenchymal transition by repressing E-cadherin [31, 32] and is implicated in cancer invasion and metastasis. It was previously demonstrated that *SNAI2* expression was induced by treating HeLa cells with IGF-1 for 30 minutes [33].

**Figure 4:**
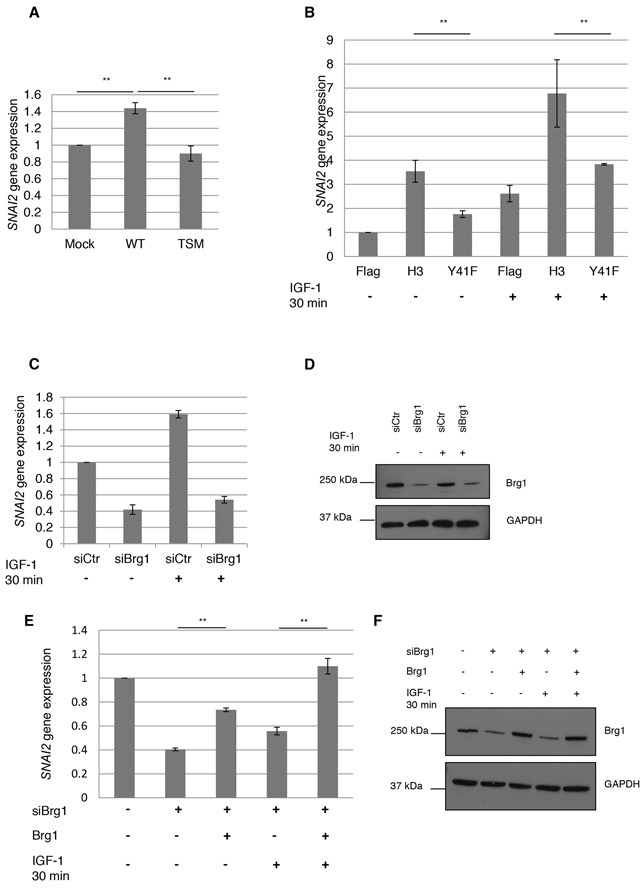
*SNAI2* gene expression by nIGF-1R and pH3Y41 **A.** *SNAI2* expression by nIGF-1R was assesed by qPCR in HeLa cells, under basal conditions, transfected with mock, *wt-IGF1R* or *tsm-IGF1R* using *SNAI2* and *GAPDH* (for normalization) primer/probes. **B.** *SNAI2* gene expression levels were determined in HeLa cells transfected with Flag, Flag H3 or Flag H3Y41F for 24 h followed by serum starvation for an additional 24 h. Serum starved cells were stimulated with 100 ng/ml IGF-1 for 0 or 30 min. **C.** *SNAI2* expression in HeLa cells, growing under basal conditions, assayed by qPCR after siRNA treatment (48 h) against Brg1 (siBrg1), or siRNA control (siCtr), followed by 30-min IGF-1 treatment. **D.** Efficacy of siBrg1 in HeLa cells was analyzed by western blotting using a Brg1 antibody. **E.** *SNAI2* expression rescue experiment. Brg1 was knocked down with siBrg1 for 24 h followed by transfection with mock or Brg1 DNA constructs for additional 24 h in HeLa cells. Transfected cells were stimulated with 100 ng/ml IGF-1 for 0 or 30 minutes and *SNAI2* expression assayed with qPCR. **F.** Transfection efficiencies were analyzed with western blot. One-way ANOVA and Tukey's HSD test were perfomed. **p<0.01. Means and s.d. are shown (n=3).

Using the same culture conditions (no IGF-1 addition) as for the PCR array, we could confirm by qPCR that *wt-IGF1R* transfected HeLa cells, compared to *tsm-IGF-1R* transfected ones, have a statistically significant increase in *SNAI2* expression, 1.44 fold compared to 0.90 fold (Figure 4A).

Next we investigated whether H3Y41 phosphorylation could increase *SNAI2* expression. HeLa cells were transfected with wt-H3 or H3Y41F constructs for 24 h followed by serum starvation for additional 24 h. Serum starved cells were then treated with IGF-1 for 0-30 min and *SNAI2* expression was analyzed with qPCR. In all cases IGF-1 treatment increased *SNAI2* expression compared to the non-treated cells (Figure 4B). This is probably explained by ligand-induced increase in phospho-nIGF-1R and nIGF-1R/H3 association (cf. Figure 1E). It is also in consistence with the findings that inhibition of phospho-IGF-1R decreases nIGF-1R/H3 association (cf. Figure 1F) and destabilizes the binding of Brg1 to histone H3 (cf. Figure 3). Also, cells transfected with *wt-H3* (treated or not treated with IGF-1) had a higher expression of *SNAI2* compared to Flag and H3Y41F transfected cells. The difference between wt-H3 and H3Y41F in this context is not unexpected as phosphorylation of tyrosine 41 seems to favor recruitment of Brg1 (cf. Figure 3A and 3B). That H3Y41F expression also increased *SNAI2* expression compared to the Flag control, although to a smaller extent, could be explained by that other modifications of Histone H3 increase *SNAI2* expression. Supplementary Figure S3 shows *SNAI2* expression in wt-IGF-1R and TM-IGF-1R (inactive IGF-1R) transfected HeLa cells, stimulated with IGF-1 for 0-30 min. Though wt-IGF-1R cells stimulated with IGF-1 significantly increases *SNAI2* expression compared to mock, the increase is not significantly higher compared to TM-IGF-1R. The p-value from Tukey's HSD test is close to p<0.05 (p=0.077). Furthermore, TM-IGF-1R did not differ significantly from the mock-control, which wt-IGF-1R did, and all results in Figure 4 provide strong evidence for connection between nIGF-1R and *SNAI2* expression.

In order to determine whether IGF-1 induced expression of *SNAI2* was dependent on Brg1, we used small interfering RNA (siRNA) to deplete Brg1. HeLa cells were transfected with siBrg1 or control siRNA (siCtr) for 48 h followed by IGF-1 stimulation for 0-30 minutes. *SNAI2* expression was determined with qPCR, Figure 4C. We did not serum starve the cells since starvation decreased the efficiency of siBrg1. In cells not stimulated with IGF-1, siBrg1 significantly reduced the expression of *SNAI2*. This reduction could not be restored by IGF-1 stimulation. Western blot confirmed good efficiency of siBrg1 (Figure 4D). To further confirm that IGF-1 induced expression of *SNAI2* requires Brg1, we performed rescue experiments. HeLa cells were transfected with siCtr or siBrg. After 24 h of siRNA transfection, the cells were transfected with either mock or Brg1 DNA constructs [34] for additional 24 h followed by stimulation with IGF-1. Figure 4E shows that overexpression of Brg1 can rescue the expression of *SNAI2* in cells with reduced levels of Brg1, even in absence of IGF-1 stimulation (third bar). Stimulation with IGF-1 further increases *SNAI2* expression (fifth bar) to the extent that is comparable to the control (first bar). Western blot confirmed good transfection efficiency (Figure 4F). These data strongly suggest that Brg1 is necessary for *SNAI2* expression.

### IGF-1R and Brg1 binds the SNAI2 promoter

**Figure 5:**
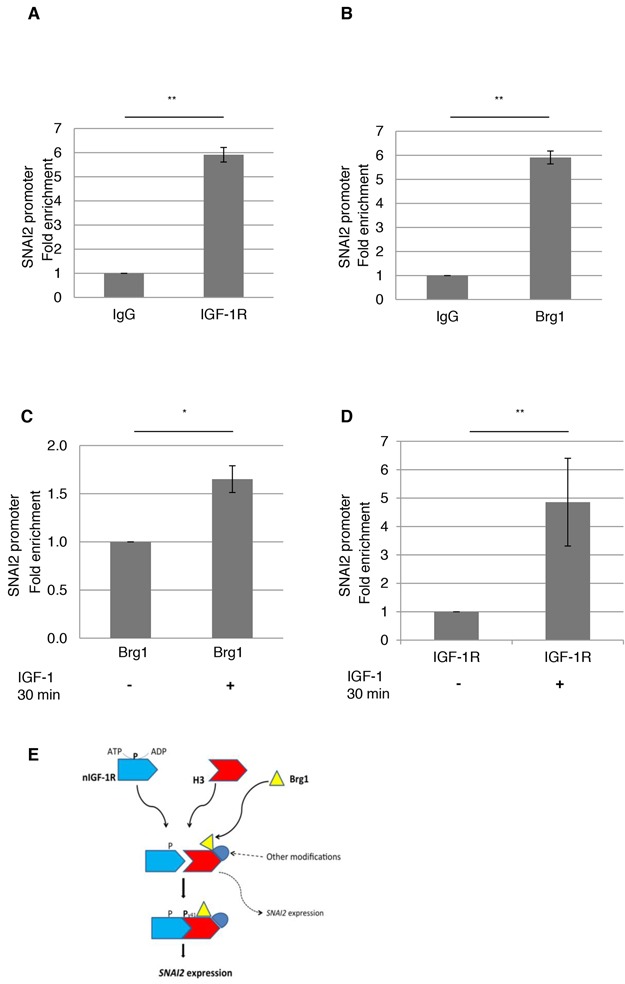
IGF1-R and Brg1 binds to SNAI2 promoter **A.** and **B.** Nuclear IGF-1R and Brg1 binding to SNAI2 promoter was assessed by ChIP-qPCR using IGF-1R or Brg1 antibodies, and anti-IgG as a negative control antibody, in HeLa cells cultured in basal conditions. **C.** and **D.** HeLa cells serum starved for 24 h followed by 0 or 30-min IGF-1 treatment were assayed for Brg1 or IGF-1R binding to SNAI2 promoter. Two-sided Student's t-test. *p<0.05, **p<0.01. Means and s.d. are shown (n=3). **E.** Schematic description of nIGF-1R interaction with Histone 3/Brg1 and its effect on *SNAI2* expression.

To further explore the role of nIGF-1R and Brg1 in *SNAI2* expression, we investigated whether they were associated with the *SNAI2* promoter. To test this, we performed chromatin IP (ChIP). Chromatin from HeLa cells cultured in basal condition was subjected to ChIP using IgG (negative control), IGF-1R or Brg1 antibodies. Co-precipitated chromatin was subjected to qPCR to evaluate enrichment of the *SNAI2* promoter. Our results show that *SNAI2* promoter is enriched in both IGF-1R and Brg1 precipitates (Figures 5A and 5B). We also investigated whether Brg1 and IGF-1R accumulation at the *SNAI2* promoter could be induced by IGF-1. HeLa cells were serum starved for 24 h and stimulated with IGF-1 for 0 or 30 min prior to Brg1 or IGF-1R pull down as previously. Figure 5C and 5D show that IGF-1 stimulation significantly increased binding of Brg1 and IGF-1R, respectively, to the *SNAI2* promoter. Figure 5E shows a schematic summary of our main findings (see also Discussion).

## DISCUSSION

Nuclear localization of IGF-1R was first reported in hamster kidney cells in 1996 [35]. Treatment of hamster kidney cells with diethylstilbestrol (a synthetic nonsteroidal estrogen) induced nIGF-1R. The authors did not provide any functional studies of nIGF-1R, nor did they explore the mechanism for the nuclear translocation of the receptor. It was not until 2010, when our lab confirmed nuclear localization of IGF-1R, that nuclear translocation of IGF-1R was shown to be dependent on IGF-1 and SUMOylation [22]. We also provided evidence that nIGF-1R was enriched at enhancer sites in genomic DNA to activate transcription of yet unknown genes. These data indicated that nIGF-1R was involved in transcriptional activation. Since our original study, numerous studies about nIGF-1R have been published, both by us as well as others [23–25, 36–39]. Interestingly, nIGF-1R is significantly more abundant in cancer cells compared to normal cells [40] and correlated with poor survival [23, 41]. It has also been reported that nIGF-1R increases cell proliferation [42] and affects therapeutic response and resistance [37, 43], suggesting that nIGF-1R is implicated in cancer. Understanding the functional role of nIGF-1R is therefore of special interest. In this study we provide data that supports the idea that nIGF-1R is involved in transcriptional activation in cancer.

Aleksic et al. have previously shown that histone H3 co-precipitated with nIGF-1R in prostate cancer cells (DU145) [23]. We could confirm this in HeLa cells and that IGF-1R binding to H3 was induced by IGF-1. The kinetics of ligand-induced nuclear translocation of IGF-1R is similar between DU145 cells and non-small cell lung cancer cells (H1299) [23, 24]. Both these cell lines, in contrast to melanoma cells (DFB) [22], exhibit quite a high nIGF-1R level after serum starvation and it increases only moderately upon IGF-1 stimulation [23, 24]. Also as shown in this study, HeLa cells have a high baseline level of and minor-modest ligand-induced increase in total nIGF-1R. However, we found that phospho-nIGF-1R was completely depleted after serum starvation and increased clearly upon stimulation with IGF-1. This was correlated with an increase in nIGF-1R binding to histone H3. To further explore whether phosphorylated nIGF-1R is important for the binding of IGF-1R to histone H3, we blocked IGF-1R phosphorylation with a specific kinase inhibitor. As a consequence of this treatment, the amount of nIGF-1R bound to histone H3 drastically decreased. Taken together, these data suggest that rather than the total level of nIGF-1R, it is the level of phospho-nIGF-1R that is important for IGF-1R to bind histone H3.

A key finding in this study is the phosphorylation of H3Y41 by nIGF-1R. It has previously been reported that Jak2 phosphorylates H3Y41 in leukemic cells [9, 28]. Y41 is localized closed to N-terminus of the first helix of histone H3 where DNA enters the nucleosome, which makes it accessible for modification [9]. When wt-IGF-1R and tsm-IGF-1R were overexpressed, pH3Y41 was significantly increased in *wt-IGF1R* transfected cells compared to *tsm-IGF1R* transfected cells. This suggests that nIGF-1R induces H3Y41 phosphorylation. The *in vitro* kinase assay also detected pH3Y41 in presence of recombinant IGF-1R, strongly indicatingthat nIGF-1R is the kinase for this phosphorylation. To strengthen our hypothesis further, we performed kinase assays using endogenous IGF-1R from HeLa nuclear extracts. As with recombinant IGF-1R, endogenous nIGF-1R induced histone H3 tyrosine phosphorylation as well. Accordingly, this is the first study providing evidence that a receptor tyrosine kinase phosphorylates histone proteins.

Given the fact that phosphorylated H3Y41 in leukemic cells is highly enriched in active genes [9, 28] and that modified histones serves as a recruitment platform for chromatin remodeling proteins [1], it was of special interest to investigate whether nIGF-1R induced phosphorylation of H3Y41 could recruit such proteins. The ATP-dependent helicase Brg1, binds to a region in proximity to H3Y41 to remodel chromatin *in vitro* [30]. We hypothesized that phosphorylated H3Y41 could serve as signal to recruit Brg1 and promote chromatin remodeling. We found that Brg1 was significantly more strongly bound to wt H3 compared to H3Y41F. These data suggest that pH3Y41 *per se* is not a requirement for the binding of Brg1 to histone H3, as it was shown previously [30]. Instead, phosphorylation of H3Y41 may stabilize Brg1 binding to histone H3 in order to promote chromatin remodeling. Because Brg1 does not harbor any phosphotyrosine binding domains (PTB/SH2), it is unlikely that Brg1 directly binds to pH3Y41. However, it is possible that the phosphorylation of H3Y41 induces a modification of nearby residues, since crosstalk between histone modifications is very common [44–47]. It is well known that phosphorylation and acetylation crosstalk occurs [44–46, 48]. pH3Y41 might promote histone H3 acetylation of a nearby lysine residue, which in turn is recognized and bound by Brg1 through its bromodomain. Brg1, bound to acetylated histone H3, is stabilized and then an efficient chromatin remodeling can take place. A direct support for a stabilizing role of nIGF-1R induced phosphorylation of histone H3 in Brg1 binding is our experiment showing that inhibition of the IGF-1R kinase decreases Brg1 binding to histone H3.

We identified *SNAI2* as a target gene for nIGF-1R. *SNAI2* is a key regulator of cell migration and epithelial-mesenchymal transition (EMT). It is a member of the E-cadherin transcriptional repressor family, which is activated by Brg1. We showed that wt H3 increases *SNAI2* compared to H3Y41F. The expression of *SNAI2* was further enhanced when cells were stimulated with IGF-1, confirming that *SNAI2* is a target gene of IGF-1R. An interesting finding was that *SNAI2* expression was significantly reduced when Brg1 was knocked down with siRNA. As loss of Brg1 can, in certain cases, be compensated by Brm [49, 50] we speculated that IGF-1 stimulation could rescue *SNAI2* expression by activating Brm or other chromatin remodeling proteins. Surprisingly, *SNAI2* expression was not restored by IGF-1. Further support for the requirement of Brg1 is the fact that the expression of *SNAI2* was rescued by overexpressing Brg1 in HeLa cells with reduced levels of Brg1. These data suggests that IGF-1 induced expression of *SNAI2* requires Brg1.

To further understand the mechanism of *SNAI2* expression, we investigated whether IGF-1R and Brg1 could bind to the SNAI2 promoter by chromatin IP. We specifically investigated a region within 1kb upstream of the transcriptional start site as several transcription factors have been reported to bind this region [51–53]. SNAI2 promoter was enriched in IGF-1R and Brg1 precipitates in basal conditions. Stimulation with IGF-1 also enriched SNAI2 promoter in IGF-1R and Brg1 precipitates. This suggests that IGF-1 stimulation induces their binding to the SNAI2 promoter to promote chromatin remodeling and gene expression. Figure 5E summarizes our major findings and hypotheses schematically; how the interaction between nIGF-1R and histone H3 causes Brg1 recruitment and *SNAI2* expression. Further studies on possible role of the nIGF-1R/H3/Brg1/SNAI2 pathway in migration and epithelial-mesenchymal transition will be of large interest.

This study highlights the importance of nIGF-1R as a transcriptional activator in cancer. Our findings may be a useful step in closer understanding of IGF-1R's role in cancer and as such may contribute to development of novel targeted strategies inhibiting cancer-specific pathways.

## MATERIALS AND METHODS

### Antibodies

IGF-1R (3027), pIGF-1R Y1135/Y1136 (3024), Flag (2368), histone H3 (9715) and Na^+^/K^+^-ATPase (3010) antibodies were from Cell Signaling Technology, Danvers, CO. IgG (H-270), Brg1 (H-88) and pan-phosphotyrosine (PY99) antibodies were from Santa Cruz Biotechnology, Dallas, TX. pH3Y41 (ab26310) antibody came from Abcam, Cambridge, UK.

### Plasmids

The plasmids containing wt human *IGF1R* and TM-IGF1R (mutated Y1131, Y1135 and Y1136) cDNA was a kind gift from Dr. Renato Baserga (Thomas Jefferson University, Philadelphia, PA). The triple SUMO mutant (TSM) IGF-1R has been described elsewhere [22]. To generate histone H3 constructs, total mRNA was first extracted with RNeasy Mini Kit (Qiagen, Hilden, Germany) and used as template for reverse transcription with VILO cDNA Synthesis Kit (Life Technologies) according to manufacturer's protocol. Histone H3 was amplified using the following primers; forward 5′- GGTTCCGCGTGGATCCATGGCTCGTACTAAACAGACAGC-3′ and reverse 5′-GTCGACCCGGGAATTCTTACGCTCTTTCTCCGCGAAT-3′. PCR product was inserted into pcDNA3.1 (+) vector with a C-terminal FLAG tag using In-Fusion Dry-Down PCR Cloning Kit (Clontech, Mountain View, CA). Proper sequence was confirmed with sequencing. Human Brg1 plasmid was a gift from Jerry Crabtree (Addgene plasmid #17873).

### Cell culture and transfections

Human cervical carcinoma (HeLa) and human leiomyosarcoma (SKUT-1) cells (both authenticated by ATCC, Manassas, VA) were cultured in DMEM and MEM respectively and supplemented with 10% fetal bovine serum. Cells were maintained at 37°C and 5 % CO_2_. Both cell lines were tested for absence of Mycoplasma with MycoAlert^TM^ Mycoplasma Detection Kit (Lonza, Basel, Switzerland). HeLa cells were transfected with indicated plasmids using Fugene HD (Promega, Madison, WI) according to manufacturer's protocol. For siRNA experiments, cells were transfected with SMARTpool: ON-Target plus Brg1 siRNA using Dharmafect 1 transfection reagent (Dharmacon, Lafayette, CO). Plasmid and siRNA transfections were carried out for 48 h.

### Co-immunoprecipitation/Western blot

Cells were lysed in lysis buffer (50 mM Tris pH 7.5, 150 mM NaCl, 1 % NP-40, 0.25 % sodium deoxycholate, 1 mM EDTA and supplemented protease and phosphatase inhibitor cocktails). After pre-clearing with Dynabeads Protein G (Life Technologies) for 1h at 4°C, the lysates were incubated with antibody and Dynabeads overnight. The immuno-complexes were washed three times with lysis buffer and eluted by boiling in LDS sample buffer (Life Technologies). Protein lysates were separated by SDS-PAGE and transferred onto a nitrocellulose membrane (GE Healthcare, Uppsala, Sweden) and detected by Western blotting using primary antibodies overnight followed by incubation with horseradish peroxidase conjugated secondary antibody and visualized with ECL detection reagents.

### Nuclear protein extraction

Nuclear proteins were extracted using the Qproteome Cell Compartment Kit (Qiagen, Hilden, Germany) according to manufacturer's protocol.

### Duolink *in situ* proximity ligation assay (PLA)

PLA enables the visualization of two interacting proteins by simultaneously labeling them with two primary antibodies. The labeled proteins are detected by PLA probes, which are secondary antibodies conjugated with oligonucleotides that are complementary to each other. If the proteins of interest are within 30-40 nm of each other, the oligonucleotides will hybridize and undergo rolling-circle amplification (RCA) in presence of a ligase and polymerase. Fluorecently labeled oligonucleotides will hybridize with the RCA product and the protein interaction is visualized as a red spot by fluorescence microscopy. HeLa cells were fixed with 4% paraformaldehyde and permeabilized with 0.1% Triton-X100 for 10 min. The cells were incubated with Flag and IGF-1R primary antibodies overnight at room temperature in a humidity chamber. PLA was conducted according to the manufacturer's protocol (OLINK Bioscience, Uppsala, Sweden). A wide-field Zeiss (Oberkochen, Germany) Axioplan2 microscope was used, at 60x. An AxioCamHR camera was used to acquire the images. All images were analyzed in AxioVision 3.1 software.

### *In vitro* kinase assay

500 ng of recombinant H3 (New England Biolabs, Ipswich, MA) was incubated with 150 ng of recombinant IGF-1R (Life Technologies) in tyrosine kinase buffer (60 mM HEPES pH 7.5, 5 mM MgCl_2,_ 5 mM MnCl_2_, 3 μM Na_3_VO_4_, 2.5 mM DTT and ATP) for 20 min at 30°C. Reaction was stopped by addition of LDS sample buffer (Life Technologies).

### Quantitative PCR

RNA was extracted with RNAeasy Mini Kit (Qiagen, Hilden, Germany) and used for reverse transcription to generate cDNA according to manufacturer's protocol. Gene expression was measured using TaqMan based *SNAI2* and *GAPDH* (for normalization) primer/probes (Life Technologies). The thermal cycling conditions were as follow, 2 minutes at 50°C, 10 minutes at 95°C, 40 cycles for 15 seconds at 95°C and 1 minute at 60°C.

### PCR Array

The RT^2^ Profiler PCR Array from Qiagen (Hilden, Germany) was utilized to identify target genes of wt IGF-1R. The kit consists of a 96-well plate with primers for cancer-related genes attached to the bottom of each well. Total RNA and reverse transcription into cDNA from HeLa cells transfected with indicated plasmids under basal conditions, as previously described, was used as template for the array.

### Chromatin immunoprecipitation

ChIP assay was performed using ChIP assay kit (Upstate Biotechnology, Lake Placid, NY) according to the manufacturer's protocol. In short, chromatin was sonicated 2 × 10 min to an average size of 300 - 800 bp. Prior to IP, 1 % of input was saved. Brg1, IGF-1R or IgG control antibodies were used for IP. Quantification of co-precipitated DNA was performed by qPCR using ABI PRISM 7500 Sequence Detection System, EpiTect Chip qPCR Primer (catalog number: GPH025770(−)01A) and RT^2^ SYBR Green Master Mix (Qiagen, Hilden, Germany). The queried site primer detects the *SNAI2* promoter (Human *SNAI2*, NM_003068.3 (−)01 kb). The cycling parameters were as followed: one cycle at 95°C for 10 min; 40 cycles of 95°C for 15 s and 60°C for 1 min.

### Statistical analysis

Immunoblots and qPCR data were normalized against their respective input controls. Means and standard deviations were calculated. One-way ANOVA, Tukey's HSD test and two-sided Student's t-test were used for statistical analysis.

## SUPPLEMENTARY FIGURES AND TABLE


