# Supplementary material for: Nuclearly translocated insulin-like growth factor 1 receptor phosphorylates histone H3 at tyrosine 41 and induces SNAI2 expression via Brg1 chromatin remodeling protein
Source: Oncotarget. 2016 Jun 2;7(27):42288–302. doi: 10.18632/oncotarget.9785 (PMC5173135; doi:10.18632/oncotarget.9785)
Supplement: Supplementary file 1 [file oncotarget-07-42288-s001.pdf]

Nuclearly translocated insulin-like growth factor 1 receptor phosphorylates histone H3 at tyrosine 41 and induces *SNAI2* expression *via* Brg1 chromatin remodeling protein

SUPPLEMENTARY FIGURES AND TABLE

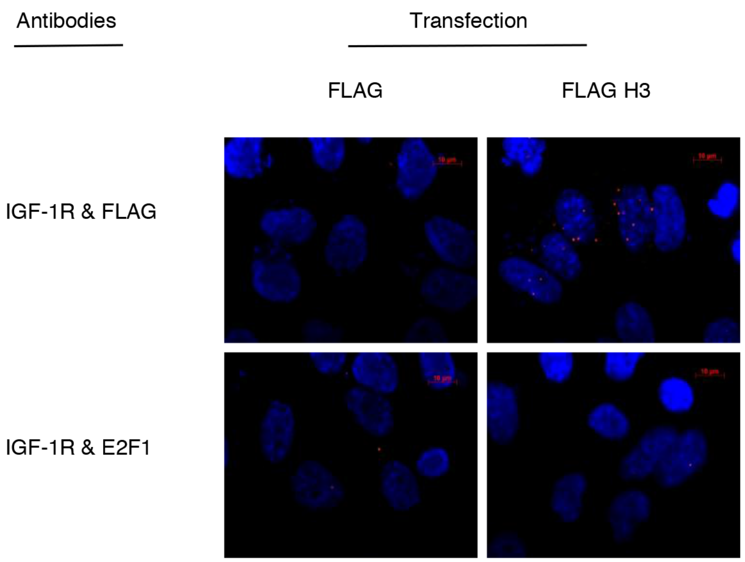

Supplementary Figure S1: nIGF-1R binds to histone H3. nIGF-1R and histone H3 binding determined with PLA (red dots) in HeLa cells transfected with an empty Flag vector (top left) or Flag H3 construct (top right). Anti-Flag and anti-IGF-1R antibodies were used. For negative control, PLA between IGF-1R and E2F1 did not show any signal (bottom panels). Cells were growing in basal conditions.

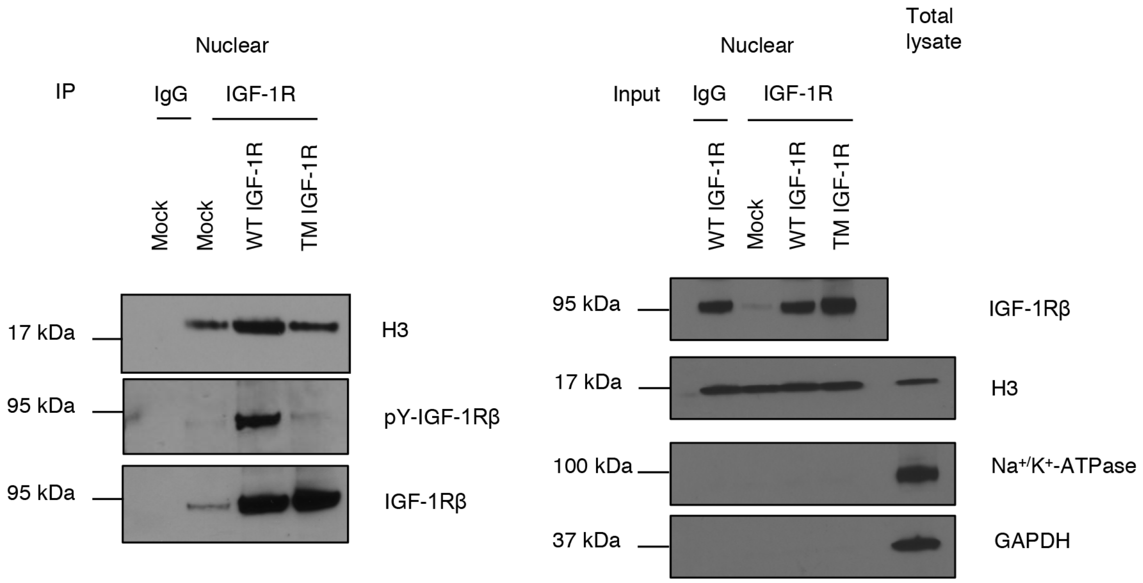

Supplementary Figure S2: IGF-1R phosphorylation is important for its binding to histone H3. HeLa cells cultured under basal conditions were transiently transfected with mock, *wt-IGF1R* or *tm-IGF1R* for 48 hours. Nuclear extracts were subjected to IP with IgG or IGF-1R antibodies and blotted for histone H3, phosphotyrosine-IGF-1R and IGF-1R (left panels). Right panels show purity of nuclear fractions.

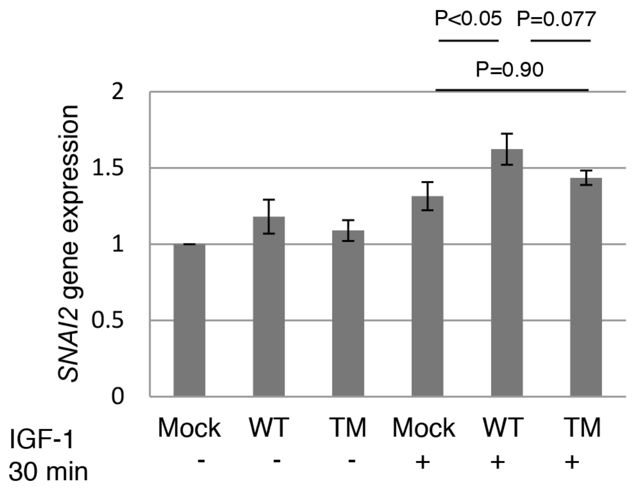

Supplementary Figure S3: *SNAI2* gene expression by wt and tm-IGF-1R. HeLa cells cultured under basal conditions were transfected with mock, *wt-IGF-1R* or *tm-IGF-1R* for 24 hours. After 24 hours, the cells were serum starved for an additional 24 hours. Serum starved cells were stimulated with 100 ng/ml IGF-1 for 0-30 minutes and *SNAI2* expression was analyzed with qPCR. One-way ANOVA and Tukey's HSD test were performed. P-values are indicated. Means and s.d. are shown (n=3).

Supplementary Table S1: Identification of nIGF-1R target genes by PCR array

	wt-IGF1R	*tsm-IGF1R*
**Gene**	**Fold change**	**Fold change**
ARNT	-1.0754	-1.7223
AURKA	-1.1051	-1.3286
BIRC3	1.2182	-1.0754
BMI1	-1.0498	-1.5638
CCND2	3.3681	5.3244
DDB2	-1.4075	-1.8561
DDIT3	2.157	1.3646
DKC1	1.2103	-1.027
E2F4	-1.0373	-1.5605
ERCC3	1.0774	-1.1268
ERCC5	1.2552	-1.2045
ETS2	-1.2203	-2.0247
FOXC2	-1.1939	-1.4475
GSC	1.6681	3.0432
MCM2	1.0559	-1.3938
MKI67	-1.2054	-1.7043
NOL3	1.114	-1.3191
PINX1	1.1587	-1.3956
POLB	1.1331	-1.3281
SKP2	1.0207	-1.3643
SNAI1	-1.0064	-2.3167
SNAI2	1.1915	-1.1338
SNAI3	1.2047	-1.5561
SOX10	-1.279	1.565
TBX2	1.4937	-1.4026
TEP1	1.2796	-1.3499
TERF1	1.6648	-1.2004
TERF2IP	1.4605	1.1349
TINF2	-1.0302	-1.3631
WEE1	2.2683	-1.3681

HeLa cells cultured under basal conditions were transiently transfected with mock, *wt-IGF1R* or *tsm-IGF1R* for 48 hours. RNA was extracted and converted into cDNA (see Materials & Methods). Gene expression was measured using the RT^2 Profiler PCR Array. The results include genes whose proteins are nuclear/nuclear-associated. Gene expression is shown as fold change compared to mock transfected cells.
